# Paraneoplastic syndromes revealing ovarian teratoma in young and menopausal women: report of two cases

**DOI:** 10.11604/pamj.2016.24.161.6743

**Published:** 2016-06-25

**Authors:** Majdouline Boujoual, Ihsan Hakimi, Farid Kassidi, Youssef Akhoudad, Nawal Sahel, Adil Rkiouak, Mohamed Allaoui, Hafsa Chahdi, Mohamed Oukabli, Jaouad Kouach, Driss Rahali Moussaoui, Mohamed Dehayni

**Affiliations:** 1 Department of Gynecology-Obstetric, University of Medicine Tangier, Military Training Hospital Med V, Rabat, Maroc; 2 Department of Gynecology-Obstetric, Military Training Hospital Med V, Rabat, Maroc; 3 Department of Internal Medicine, Military Training Hospital Med V, Rabat, Maroc; 4 Department of Pathology, Military Training Hospital Med V, Rabat, Maroc

**Keywords:** Paraneoplastic syndrome, ovarian teratoma, diagnosis, management

## Abstract

Paraneoplastic syndromes are a heterogeneous group of clinical and biological manifestations caused by underling neoplasms. They can reveal ovarian teratoma which express neuroendocrine proteins, or contain mature or immature neural tissue inducing an autoimmune response. The etiological investigation is then crucial to early identification of the tumor in order to optimize the prognosis and to limit neurological sequelae. In case of ovarian teratoma, management is essentially based on surgical resection sometimes associated with immunotherapie. We report two new cases of ovarian teratoma revealed by paraneoplastic syndromes in young and menopausal woman.

## Introduction

Paraneoplastic syndromes are a heterogeneous collection of manifestations caused by underlying neoplasm [1]. It can proceed, occur with, or develop after a malignancy [2], affecting patients of all ages [3] and are often misdiagnosed [4]. Actually, major advances in the management include the discovery and improved characterization of these syndromes, detection of new anti-neuronal antibodies and the use of CT and PET scan to reveal the associated tumors at an early stage [5] in order to improve prognosis [6]. Thus, awareness of paraneoplastic syndromes is important for various practitioners, including both neurologists and gynecologists [1].

## Patient and observation

### Case N°1

**Figure 1:**
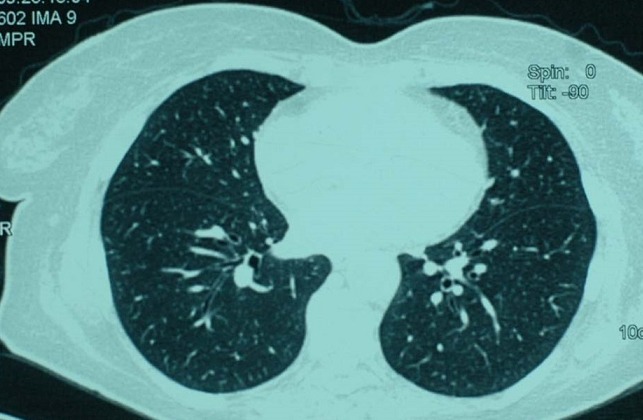
Thoracic CT revealing infra centimetric pulmonary nodules

**Figure 2:**
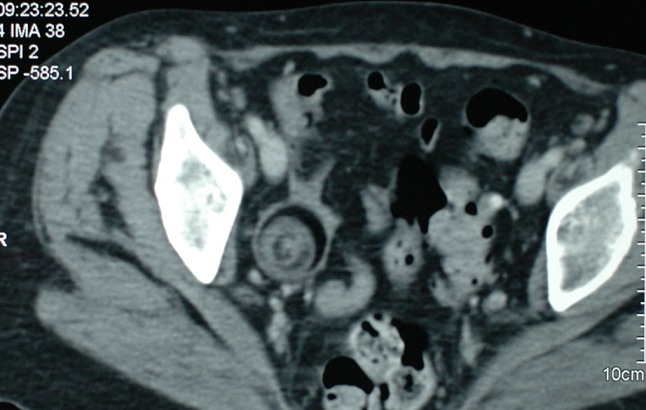
Pelvic CT showing dermoid cyst of the left ovary measuring 36 x 37 mm

**Figure 3:**
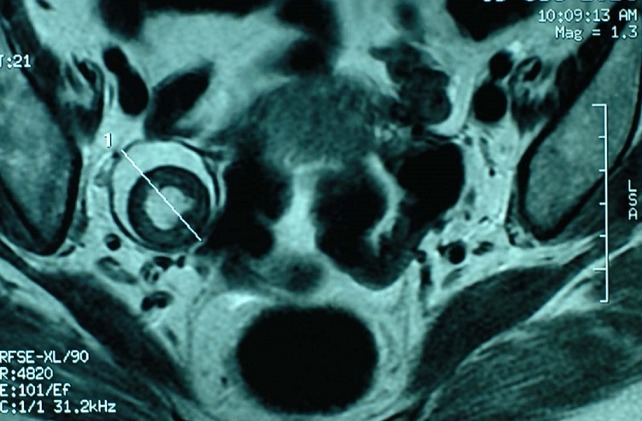
Pelvic MRI confirming dermoid cyst of the left ovary

**Figure 4:**
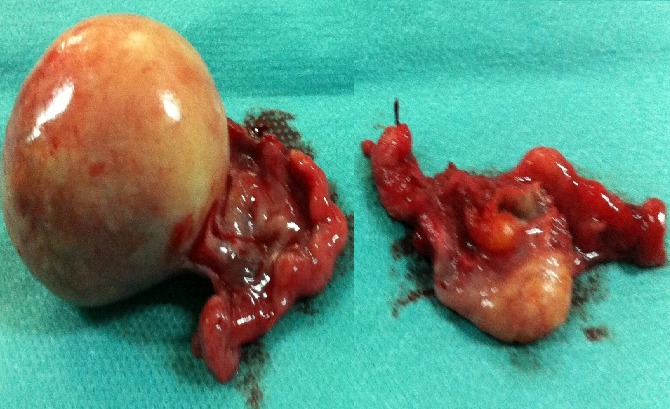
Macroscopic appearance of the resected bilateral oophorectomy

**Figure 5:**
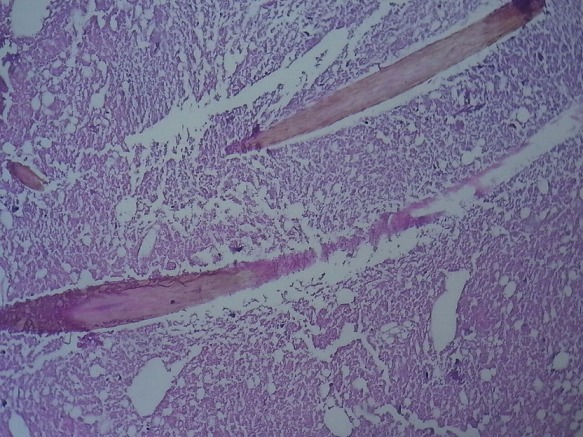
Histopathology of mature dermoid cyst (HEX40) showing microscopic appearance of hair sections

A 60-year-old menopausal female admitted to internal medicine service for myalgia and generalized weakness with weight loss of 8 kg in 2 months. Her clinical examination found a myogenic syndrome with hypotonia, muscle weakness predominant in the lower limbs and associated to functional impairment with dyspnea stage III. Biological investigation showed cytolysis with CPK: 9000 U/l, LDH: 731 U/L, AST: 179 U/L, ALT: 164 U/L and TP: 87%. Serological examination was negative including viral hepatitis A, B, C, HIV, EBV and CMV. Thyroid and parathyroid function, as immunological tests (anti- nuclear, anti -mitochondrial, anti - smooth muscle and anti LKM1) were all normal. Tumor markers showed a normal rate of AFP and ACE while the CA125 and CA19-9 were increased 2x normal and CA15-3 was 5x normal. Liver biopsy showed chronic hepatitis with a score of Metavir A1F0 without injury or tumor specific inflammatory. A paraneoplastic polymyositis was so suspected. However, electromyography and muscle biopsy didn’t show any sign of myositis. Thoraco abdominopelvic CT objectified infra centimetric pulmonary nodules with left dermoid cyst of the left ovary measuring 36 x 37 mm (Figure 1, Figure 2) which was confirmed by pelvic MRI (Figure 3). Otherwise, bronchoscopy has not objectified suspicious endobronchial lesion, the AFB in sputum was negative and the PET scan confirmed the absence of suspicious pathological fixation. The patient was put under corticosteroids (Prednisone 60mg /day with bolus of Solumedrol), and underwent an exploratory laparotomy with bilateral oophorectomy (Figure 4). Pathological study confirmed a mature dermoid cyst (Figure 5). Postoperatively, biological improvement was achieved despite the persistence of residual muscle deficit. The patient was so put on immunosuppressive treatment with bolus of Endoxan (1g/month) associated with Immunoglubuline (2g/kg/bolus), due to the partial answer (CPK: 1500 U/L). Clinical and biological improvement (resumption of physical activity, autonomy and decreased CPK to 600U/L) were so obtained, and thena relay by immunomodulatory was made after the 6th bolus of Endoxan. The patient has so well evolved, and is currently under corticosteroids regression.

### Case N°2

**Figure 6:**
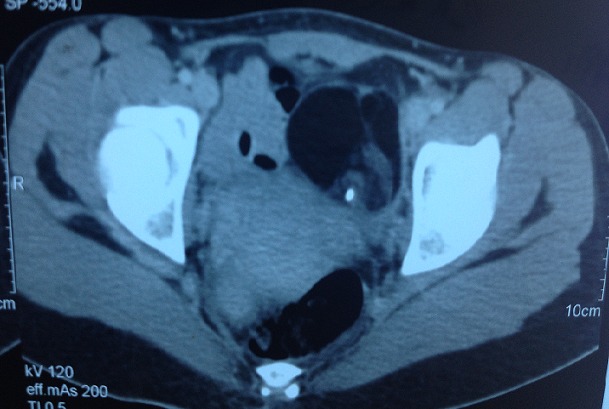
Pelvic CT showing ovarian teratoma measuring 66x 53 mm with lobulated contours, fat density and calcifications

**Figure 7:**
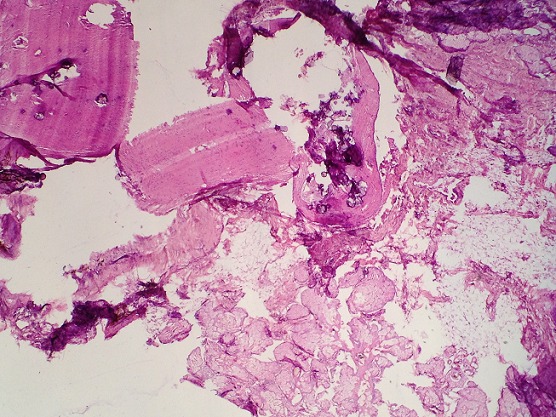
Histopathology of mature teratoma with Skin covering with pilosebaceous glands, adipose tissue and brain tissue (HEX50)

31-year -old female without a past medical history, admitted to neurological service for management of balance and walking disorder lasting for 3 years without signs of intracranial hypertension or other extra neurological signs. Her neurological examination showed a stato- kinetic cerebellar syndrome. The cerebral MRI revealed a cerebellar atrophy predominant at vermis without under cortical lesion. There was no inflammatory syndrome and cerebrospinal fluid analysis was normal. As part of etiological investigation a thoracoabdominopelvic CT objectified ovarian teratoma measuring 66/53 mm with lobulated contours, fat density and calcifications (Figure 6). The paraneoplastic cerebellar syndrome origin was suspected. The patient underwent laparoscopic cystectomy with biopsy of the contralateral ovary. Histological analysis showed a mature teratoma with skin covering pilosebaceous glands, adipose tissue and brain tissue (Figure 7). Good clinical improvement was obtained after surgery without further therapy.

## Discussion

**Definition:** Paraneoplastic syndromes (PS) are remote effects of cancer caused neither by invasion of the tumor or its metastases nor by infection, ischemia, metabolic and nutritional deficits, surgery, or other forms of tumor treatment [3]. They may precede the diagnosis [7], or herald the recurrence or a second tumor [3]. Actually, numerous types of paraneoplastic antibodies have been described. Even so, their absence cannot rule out the diagnosis of PS [4].

**Incidence:** PS are rare occurring in less than 0.01% of patients with cancer [4]. Benignsteratomas have been associated with a variety of paraneoplastic syndromes including encephalitis, paraneoplastic limbic encephalitis, opsoclonus-myoclonus syndrome, seronegative polyarthritis, tenosynovitis and autoimmune hemolytic anemia [1, 8].

**Etiopathogenesis:** PS are believed to be caused by the excessive production or depletion of bioactive substances, growth factors, hormones, or by antigen-antibody interactions with an aberrant host response to various types of cancer [2]. In case of ovarian teratoma, the hypothesis of an autoimmune pathophysiology [9] is supported by its composition of ectoderm; mesoderm and endoderm which could induce an autoimmune response [5, 8].

**Diagnosis and identification of the tumor:** Once a paraneoplastic diagnosis has been suspected, rapid identification of the tumor becomes essential but may be difficult since the tumors initially may be histologically small and localized [3, 4], as the case of ovarian teratoma [10]. One attitude is restricted to patient interrogation, complete physical examination [11] with additional oriented examination according to specific symptoms as our case of paraneoplastic cerebellar ataxia [11]. In fact, CT and MRI studies demonstrate cerebellar atrophy in the later stages of the disease [4], while the anti-bodies Anti-NMDAR encephalitis are excellent predictors of a better response to immunotherapy [12]. The other attitude includes more a thoracoabdominopelvic CT scan, gastrointestinal tract, and bronchial endoscopic explorations, mammogram, bone marrow biopsy, study of circulating lymphocyte subpopulations, and serum immunoelectrophoresis [11]. While whole-body fluoride oxyglucose positron emission tomography is recommended when conventional imaging has failed or lesions are difficult to biopsy [4, 5]. If no malignancy is revealed during, surgical exploration and removal of pelvic organs may be warranted, particularly in postmenopausal women [4].

**Treatment:** selection of a treatment plan depends on the clinical severity, rate of progression, and risk of side effects [13].

**Tumor removal:** Appears to have the best effect on neurological outcome and prevent permanent neurologic sequelae [1]. In fact, it should decrease the antigenic challenge and the risk of tumor growth or metastasis [13].

**Immunotherapy:** Has been demonstrated to stop the paraneoplastic neurological deterioration including plasma exchange, immunoadsorption, steroids, and intra venous Immunoglobulins [3].

**Evolution- prognosis:** Early diagnosis is important since patients can recover with immunotherapy and tumor resection [14]. In fact, some neurological PS are monophasic reach a plateau of severity and improve. Others are progressive and fatal [13], leaving the patients severely debilitated within weeks to months. However, slow progression, relapses or a benign course do not exclude the diagnosis [4]. In our cases, the 2^nd^ patient improved dramatically after resection, while the 1^nd^ had complete recovery after surgical resection and immunotherapy.

## Conclusion

The two cases confirm diagnosis difficulty of this association. In fact, in spite of their rarity, paraneoplastic syndromes can reveal ovarian teratomas. Their management is based on identification and resection of the tumor, optionally combined toimmunotherapy. Their prognosis is related to the risk of irreversible sequelae. Hence the interest of their early recognition with clinical, biological and radiological monitoring.
